# Chronic Kidney Disease-Associated Defect in Humoral Immune Response Is Driven by Inflammation

**DOI:** 10.3390/toxins18020104

**Published:** 2026-02-19

**Authors:** Maxime Espi, Xavier Charmetant, Floriane Fusil, Cyrille Mathieu, Marie Legras, Caroline Pelletier, Griet Glorieux, Christophe Soulage, Laetitia Koppe, Olivier Thaunat

**Affiliations:** 1 CIRI-Centre Intégratif de Recherche en Immunologie, INSERM U1111, Université Claude Bernard Lyon I, CNRS UMR 5308, Ecole Normale Supérieure de Lyon, Université de Lyon, 69007 Lyon, France; 2 Université Claude Bernard Lyon I, 69100 Villeurbanne, France; 3 Department of Nephrology, Dialysis and Clinical Nutrition, Hospices Civils de Lyon, Hopital Lyon Sud, Oullins-Pierre Bénite, 69495 Lyon, France; 4 Department of Transplantation, Nephrology and Clinical Immunology, Hospices Civils de Lyon, Edouard Herriot Hospital, 69003 Lyon, France; 5 Department of Nephrology, Hypertension and Dialysis, Hospices Civils de Lyon, Edouard Herriot Hospital, 69003 Lyon, France; 6 CarMeN, INSERM U1060, INRA U1397, Université Claude Bernard Lyon 1, Oullins-Pierre Bénite, 69310 Lyon, France; 7 Department of Internal Medicine and Pediatrics, Nephrology Unit, Ghent University Hospital, 90000 Gent, Belgium

**Keywords:** inflammation, hemodialysis, chronic kidney disease, immune deficit, humoral dysfunction, vaccines

## Abstract

Advanced chronic kidney disease (CKD) is associated with impaired humoral immunity, contributing to increased infection-related mortality and suboptimal vaccine responses, as notably observed during the COVID-19 pandemic. CKD is also marked by the accumulation of uremic toxins, but whether they directly influence T and B cell functionality remains unclear. In this translational study, we integrated clinical and biological data from 106 CKD patients with mechanistic insights from in vitro and in vivo murine models to identify the mechanisms underlying CKD-associated defects in humoral responses against T cell-dependent antigens. Contrary to our initial hypothesis, indoxyl sulfate—despite its known ability to activate Aryl hydrocarbon Receptor signaling in monocytes—did not directly impair T–B cell cooperation in coculture assays. Similarly, plasma levels of ten major uremic toxins showed no correlations with vaccine-induced antibody titers in patients. Instead, systemic inflammation emerged as the primary driver of defective humoral immunity. Murine models further confirmed that inflammation, rather than uremia alone, induces lymphopenia, disrupts lymphoid architecture, and ultimately impairs antibody production. These findings indicate that CKD-associated inflammation, rather than a direct effect of uremic toxins on adaptive immune effectors, underlies humoral immune dysfunction in CKD. Targeting inflammation may, therefore, offer a promising strategy to improve vaccine efficacy and reduce infection-related complications in this vulnerable population.

## 1. Introduction

Chronic kidney disease (CKD), which results from structural or functional abnormalities of the kidneys, is a progressive disorder that can culminate in end-stage kidney disease (ESKD), which requires renal replacement therapies, including chronic hemodialysis.

In a recent analysis focused on adults aged 20 years and older over the period from 1990 to 2023 in 204 countries and territories, global age-standardized prevalence of CKD in adults was 14·2%, a relative rise of 3·5 from 1990 [1]. Over the past three decades, fueled by population aging and the rising prevalence of metabolic disorders, CKD has become a leading global cause of mortality and one of the few non-communicable diseases with an increasing death rate [1,2].

In addition to its substantial impact on healthcare systems, CKD profoundly compromises both patient survival and quality of life, due to its associated complications [3]. Among these complications, infection-related mortality occupies a prominent place. Multiple large epidemiologic studies have shown that patients with end-stage kidney disease (ESKD) on dialysis experience higher sepsis-related mortality than the general population. In U.S. registry data, annual sepsis-attributable death rates in dialysis patients were approximately 100–300-fold greater than in the general population, even after age, race, and diabetes mellitus stratification, where rates remained nearly 50-fold higher [4]. These disparities persisted when accounting for competing causes of death, with sepsis mortality still 30–45-fold above expected general population rates [4]. This increased vulnerability was dramatically highlighted during the COVID-19 pandemic, when patients with reduced kidney function experienced disproportionately high rates of severe disease and mortality [5].

Vaccination is the most efficient and cost-effective strategy to protect this vulnerable population. However, CKD patients exhibit impaired vaccine-induced immunity, particularly against protein (i.e., T cell-dependent) antigens. Both poor seroconversion rates and rapid waning of vaccinal response have been reported after vaccination against hepatitis B, diphtheria, tetanus toxin and the receptor-binding domain (RBD) of the spike glycoprotein of SARS-Cov2 [5,6,7]. While additional vaccine doses may partially restore immunogenicity in some cases [8,9], elucidating the mechanisms responsible for impaired humoral immunity remains essential for developing more effective prophylactic strategies in CKD patients.

Regardless of the primary disease responsible for kidney destruction, CKD patients share a remarkably similar comorbidity profile. This commonality has been attributed to the accumulation of uremic toxins—solutes that are normally excreted by the kidneys, but, when retained, disrupt normal cellular physiology [10]. Several of these toxins have been proposed to directly impair lymphocyte count and/or function [11,12,13]. Among them, indoxyl sulfate (IS)—a tryptophan-derived metabolite—has emerged as a potent endogenous ligand of the aryl hydrocarbon receptor (AhR), a cytoplasmic transcription factor [14]. The free (non-protein-bound) fraction of IS activates AhR in endothelial cells, contributing to CKD-associated cardiovascular complications [15]. Interestingly, AhR signaling in B cells has been shown to inhibit B cell receptor signaling, proliferation, and class-switch recombination [16]. In T cells, AhR activation promotes the differentiation of regulatory phenotypes that suppress adaptive immune responses [17,18].

Because B–T cell cooperation within germinal centers (GCs) is essential for the generation of high-affinity, class-switched immunoglobulins against protein antigens, we hypothesized that activation of AhR by free IS in CKD patients contributes to their defective vaccine-induced humoral responses. To test this hypothesis, we conducted a translational study combining the analysis of patient samples with mechanistic in vitro and in vivo models.

## 2. Results

### 2.1. End-Stage Kidney Disease Patients Exhibit an Impaired Humoral Response to a T-Dependent Antigen

**Figure 1:**
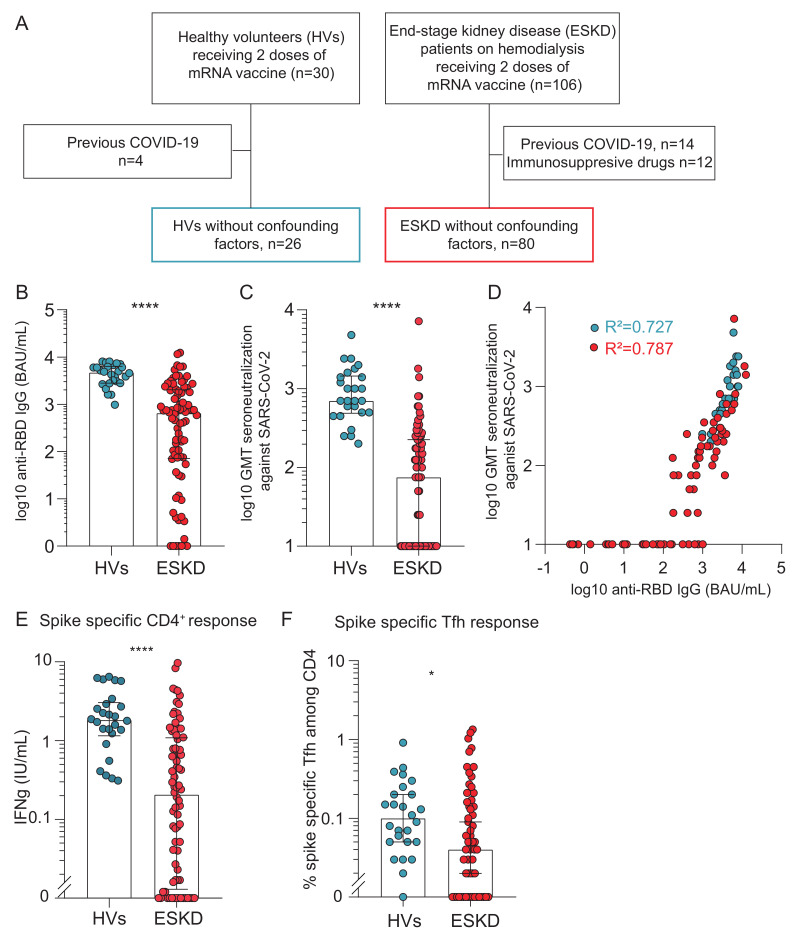
Comparison of the humoral and cellular responses to SARS-CoV-2 vaccination in healthy volunteers (HVs) and patients with end-stage kidney disease (ESKD). (**A**): Flowchart of the study cohort. (**B**–**F**): Individual values measured 10–14 days after the second vaccine dose for HVs (blue) and ESKD patients (red). (**B**): Anti-RBD IgG titers. (**C**): Log10 of the geometric mean titer of serum neutralizing SARS-CoV-2-induced cytotoxicity in target cells. (**D**): The strength of the correlation between anti-RBD IgG titers and serum neutralization capacity in HVs and ESKD patients was evaluated with r^2^: coefficient of determination. (**E**): Interferon-γ release assay quantifying spike-specific CD4^+^ T cell responses. (**F**): Percentage of spike-specific T follicular helper (Tfh) cells among CD4^+^ T cells, measured by flow cytometry. The Mann–Whitney test was used for all comparisons. *p* < 0.05 (*), *p* < 0.0001 (****). Abbreviations: HVs, healthy volunteers; ESKD, end-stage kidney disease, patients on maintenance hemodialysis; RBD, receptor-binding domain of the spike glycoprotein; BAU, binding arbitrary unit; GMT, geometric mean titer; Tfh, T follicular helper cell.

Mass SARS-CoV-2 vaccination campaigns provided a unique opportunity to characterize T cell-dependent humoral immunity in patients with CKD. We compared the vaccine-induced antibody response in 106 patients with ESKD and 30 healthy volunteers (HVs), each receiving two doses of mRNA-based SARS-CoV-2 vaccine 21 ± 2 days apart (Figure 1A). To minimize confounding factors, individuals with prior SARS-CoV-2 infection (4 HVs and 14 ESKD patients) or receiving immunosuppressive therapy (12 ESKD patients) were excluded (Figure 1A). Immune responses were analyzed 10–14 days after the second dose. The demographic and clinical characteristics of the 26 HV and 80 ESKD participants included in the final analysis are summarized in Supplementary Table S1.

Anti-RBD IgG titers were markedly lower in ESKD patients than in HVs (651 (71–2045) vs. 4728 (2813–6390) BAU/mL; *p* < 0.0001; Figure 1B). Similarly, serum neutralizing activity, assessed by cytopathic effect assay on live target cells, was substantially reduced in ESKD patients (75 (0–225) vs. 700 (487–1450); *p* < 0.0001; Figure 1C). As previously reported, anti-RBD IgG titers strongly correlated with neutralizing capacity, following an identical relationship in both groups (both *p* < 0.0001, *R*^2^ = 0.727 for HVs; *R*^2^ = 0.787 for ESKD patients; Figure 1D). These findings demonstrate that the humoral response in ESKD patients is quantitatively impaired, but qualitatively preserved, as evidenced by the conserved relationship between anti-RBD IgG titers and serum neutralizing activity.

Given that the efficient generation of high-affinity antibodies against T cell-dependent antigens (such as the RBD protein) rely not only on B cells, but also on effective T–B cell cooperation within GCs [19], we next examined SARS-CoV-2-specific CD4^+^ T cell responses. Interferon-γ release following stimulation with spike peptide pools was markedly higher in HVs than in ESKD patients (1.8 (1.2–3.0) vs. 0.21 (0.01–1.1) IU/mL; *p* < 0.0001; Figure 1E). Similarly, the frequency of spike-specific circulating follicular helper T (Tfh) cells among CD4^+^ T cells was significantly lower in ESKD patients (0.04 (0–0.16)% vs. 0.10 (0.05–0.21)%; *p* = 0.04; Figure 1F).

Collectively, these results confirm that ESKD patients display a profound quantitative defect in the humoral response to T cell-dependent antigens, likely reflecting impaired germinal center formation and T–B cell collaboration.

### 2.2. Indoxyl Sulfate Does Not Impair T–B Cooperation In Vitro

**Figure 2:**
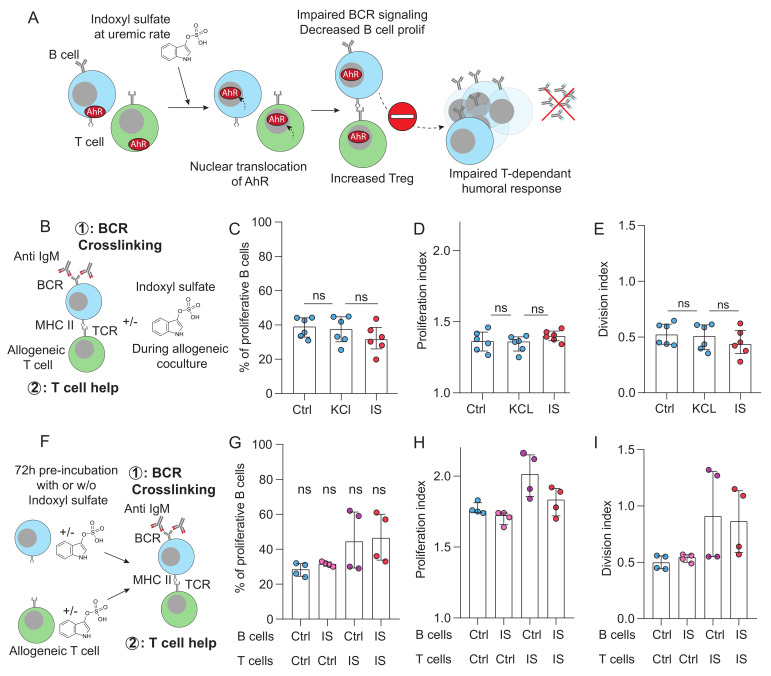
Impact of indoxyl sulfate on CD4^+^ T–B cell collaboration in vitro. (**A**): Schematic representation of the working hypothesis. In ESKD patients, exposure of B cells (LB) and/or CD4^+^ T cells (LT) to uremic concentrations of free indoxyl sulfate (IS) triggers AhR translocation within these cells, thereby impairing T–B collaboration, disrupting the germinal center reaction, and ultimately reducing antibody production. (**B**): Schematic representation of the co-culture model used to emulate germinal center-like interactions between CD4^+^ T cells and B cells in vitro. (**C**–**E**): Effects of IS on B cell proliferation: (**C**), number of proliferating B cells; (**D**), average number of divisions per proliferating B cell; and (**E**), total number of divisions per B cell. Results are shown for two control conditions (blue) and for cultures performed in the presence of IS (red). Five independent experiments. Kruskall–Wallis test. (**F**): Schematic representation of the second co-culture model designed to assess the impact of 72H IS exposure on T cells and/or B cells prior to co-culture. (**G**–**I**): B cell proliferation outcomes in the second model: (**G**), number of proliferating B cells; (**H**), average number of divisions per proliferating B cell; and (**I**), total number of divisions per B cell. Results are stratified according to which cell population(s) were pre-exposed to IS. Four independent experiments. Kruskall–Wallis test. Abbreviations: AhR, aryl hydrocarbon receptor; BCR, B cell receptor; MHC, major histocompatibility complex; TCR, T cell receptor; ns, not significant.

We hypothesized that the GC defect observed in ESKD patients could result from the accumulation of IS, a protein-bound uremic toxin known to act as a potent AhR ligand [14]. Upon activation, AhR translocates to the nucleus and regulates gene expression. In B cells, AhR activation dampens B cell receptor (BCR) signaling, proliferation, and class-switch recombination [16], whereas, in T cells, it promotes regulatory phenotypes that suppress immune responses [17,18] (Figure 2A).

To test our hypothesis, a coculture model mimicking the T–B interactions occurring in the GC reaction was used [19,20]. Briefly, purified B cells were stimulated through BCR crosslinking using anti-IgM monoclonal antibodies (signal 1) and cocultured with non-preactivated allogeneic CD4^+^ T cells. B cell proliferation was monitored over six days by CellTrace Violet dilution. In this system, allogeneic B cells activate a fraction of CD4^+^ T cells through direct TCR recognition of intact allogeneic HLA molecules, leading to T cell activation and upregulation of costimulatory signals. This T cell-derived help provides the second activation signal (signal 2), which is strictly required—together with BCR engagement—for efficient B cell proliferation (Supplementary Figure S1A,B).

Since only unbound IS exerts biological activity, we quantified both total and free IS levels in the serum of 74 ESKD patients using UPLC (Supplementary Figure S1C). Free IS levels correlated moderately with total IS concentrations (*p* < 0.001; R^2^ = 0.55; Supplementary Figure S1D). In standard culture medium, supplementation with 40 µM total IS yielded ~25 µM free IS that reflected the upper range observed in ESKD patients (Supplementary Figure S1E–G). Cocultures were, therefore, conducted in the presence of indoxyl sulfate (IS group, red) or in its absence (controls: culture medium alone or osmolar control with 40 µM KCl) (Figure 2B).

The presence of IS did not affect the proportion of proliferating B cells, the mean number of divisions per proliferating cell (proliferation index), or the total number of divisions (division index) (Figure 2C–E). To exclude the possibility that IS effects required prolonged exposure, additional coculture experiments were performed after pre-incubating B cells, T cells, or both with IS (Figure 2F). None of these conditions altered B cell proliferation (Figure 2G–I). Notably, preincubation of T cells with IS even tended to slightly enhance B cell proliferation, further arguing against a direct inhibitory effect of IS on T–B cooperation.

### 2.3. Lack of Correlation Between Uremic Toxin Levels and Vaccine-Induced Humoral Response in ESKD Patients

**Figure 3:**
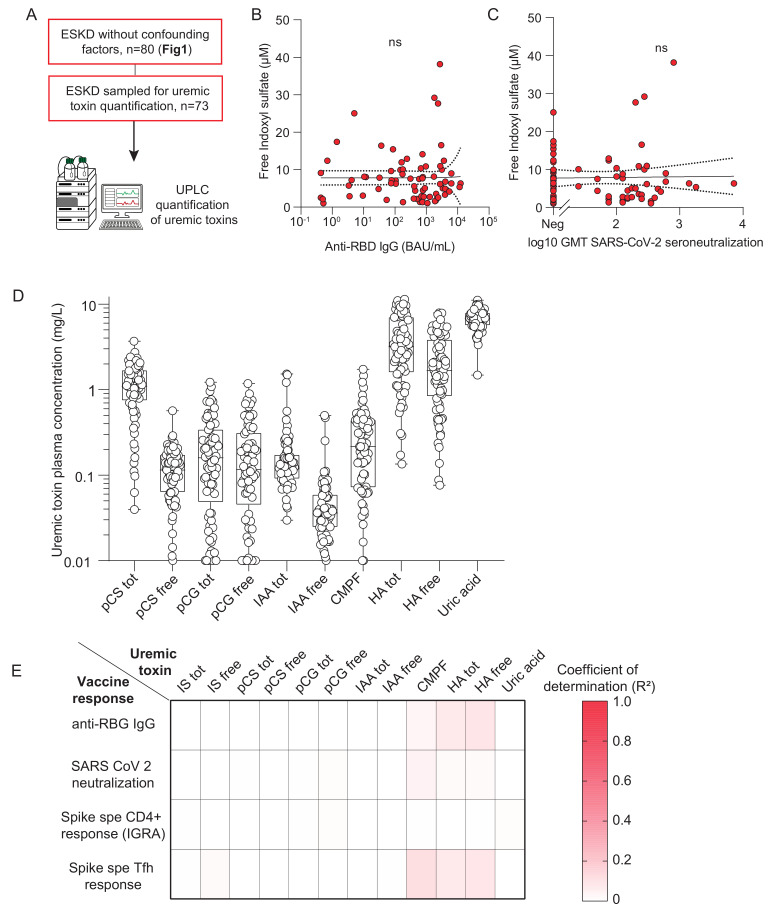
Relationship between uremic toxin levels and the humoral response to SARS-CoV-2 vaccination in ESKD patients. (**A**): Flowchart of the study cohort. (**B**,**C**): Associations between the concentration of free indoxyl sulfate (µM) and (**B**), peak anti-RBD IgG titers (BAU/mL) or (**C**), serum neutralization capacity. (**D**): Serum concentrations of ten uremic toxins—including IS (indoxyl sulfate), pCS (p-cresyl sulfate), pCG (p-cresyl glucuronide), IAA (indole acetic acid), CMPF (3-carboxy-4-methyl-5-propyl-2-furanpropionic acid), and HA (hippuric acid)—were quantified in ESKD patients using ultra high-performance liquid chromatography (UPLC). Individual values are shown. (**E**): Correlations between the concentration of each uremic toxin and four parameters of the humoral response to SARS-CoV-2 vaccination were assessed: anti-RBD IgG titers, serum neutralization capacity, the number of spike-specific CD4^+^ T cells measured by IGRA, and the percentage of spike-specific Tfh cells among CD4^+^ T cells measured by flow cytometry. The heatmap displays determination coefficients, ranging from white (R^2^ = 0) to red (R^2^ = 1).

To confirm that IS accumulation is not associated with defective vaccine responses in vivo, we analyzed the 74 patients of the ESKD cohort for which both humoral immune parameters (Figure 1) and plasma free IS concentrations (Supplementary Figure S1D) were available (Figure 3A). Free IS levels varied widely among patients (median 6.3 (2.9–10.0) µM). However, no correlation was observed between free IS concentrations and either anti-RBD IgG titers (Figure 3B) or serum neutralization capacity (Figure 3C).

To assess whether other uremic toxins might contribute, we quantified 12 additional solutes by UPLC, including total and free fractions of p-cresyl sulfate, p-cresyl glucuronide, indole-3-acetic acid, CMPF, hippuric acid, and uric acid (Figure 3D). Correlation analyses between each toxin and four parameters of the vaccine-induced response (anti-RBD IgG, serum neutralization capacity, spike-specific CD4^+^ T cells, and spike-specific Tfh cells) yielded 48 associations in total. None of them reached statistical significance (Figure 3E).

Together, these data indicate that the accumulation of uremic toxins, including IS, does not directly account for the impaired vaccine-induced humoral response observed in ESKD patients.

### 2.4. Normal T-Dependent Humoral Response in Murine Models of Chronic Kidney Disease

**Figure 4:**
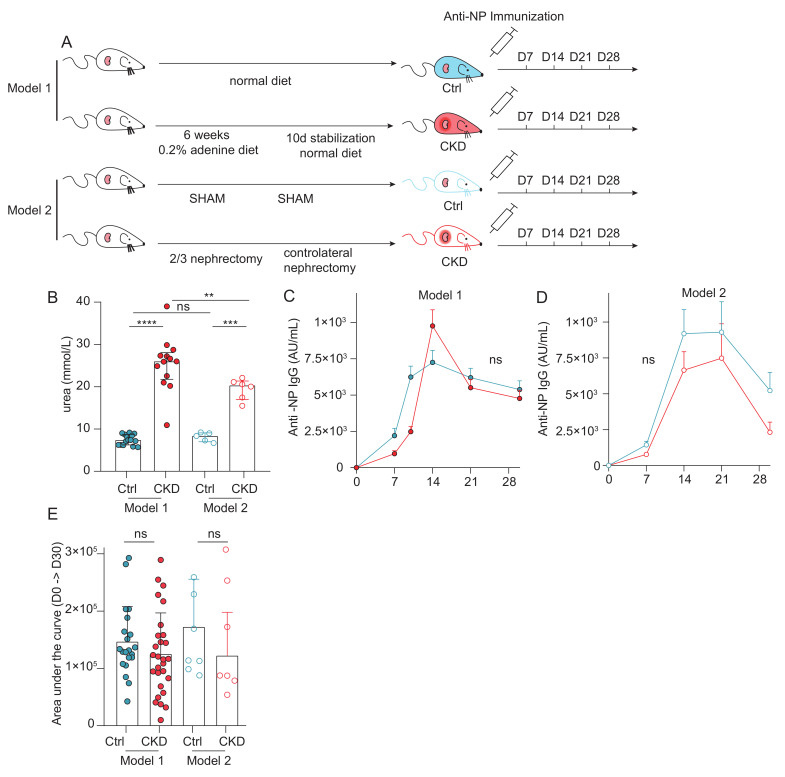
Humoral responses in murine models of chronic kidney disease. (**A**): Schematic overview of the experimental design. Color coding: control mice (blue; filled circles: control model 1; open circles: control model 2) and CKD mice (red; filled circles: CKD model 1; open circles: CKD model 2). (**B**): Comparison of urea levels (mM) between control and CKD groups. Kruskal–Wallis test. (**C**–**E**): Anti-NP IgG responses were monitored every 7 days for 4 weeks following NP–CT immunization. (**C**,**D**): Kinetics of anti-NP IgG titers in control vs. CKD mice for model 1 (**C**) and model 2 (**D**). (**E**): Area under the curve (AUC) of anti-NP IgG responses for control and CKD mice in model 1 (left) and model 2 (right). Student’s *t*-test. Abbreviations: NP, (4-hydroxy-3-nitrophenyl)acetyl hapten; CKD, chronic kidney disease; AU, arbitrary units. Statistical significance: ns, *p* > 0.05; **, *p* < 0.01; ***, *p* < 0.001; ****, *p* < 0.0001.

Because isolating in patients the effect of uremic milieu from multiple confounders is challenging, we turned to murine models of CKD to determine whether uremia alone could account for the impaired humoral response. Two well-established CKD models were used (Figure 4A). In the first model, CKD was induced by feeding mice an adenine-enriched diet (0.2%, *w*/*w*) for six weeks, followed by a return to standard chow to stabilize kidney impairment. Age-, sex-, and strain-matched animals maintained on a normal diet were used as controls. In the second model, CKD was surgically induced by 5/6 nephrectomy, consisting of the removal of two-thirds of the right kidney, followed one week later by the nephrectomy of the contralateral kidney. Sham-operated mice, subjected to anesthesia and surgery without nephrectomy, served as controls for the surgical CKD model.

Both CKD models displayed elevated blood urea levels compared with their respective controls, but higher levels were observed in the adenine model (Figure 4B). CKD and control mice were then immunized subcutaneously with the model T-dependent antigen NP–cholera toxin (NP–CT), and serum anti-NP IgG responses were monitored weekly for four weeks. Both models showed similar kinetics, with peak titers at days 14–21, followed by a decline at day 28 (Figure 4C,D). No significant differences were observed between CKD and control mice in either model. Area under the curve (AUC) analyses confirmed the absence of any global reduction in humoral response in CKD animals (Figure 4E).

Together with human data (Figure 1, Figure 2 and Figure 3), these findings indicate that CKD alone is insufficient to reproduce the humoral defect associated with ESKD.

### 2.5. Systemic Inflammation Is Associated with the Humoral Defect in ESKD

**Figure 5:**
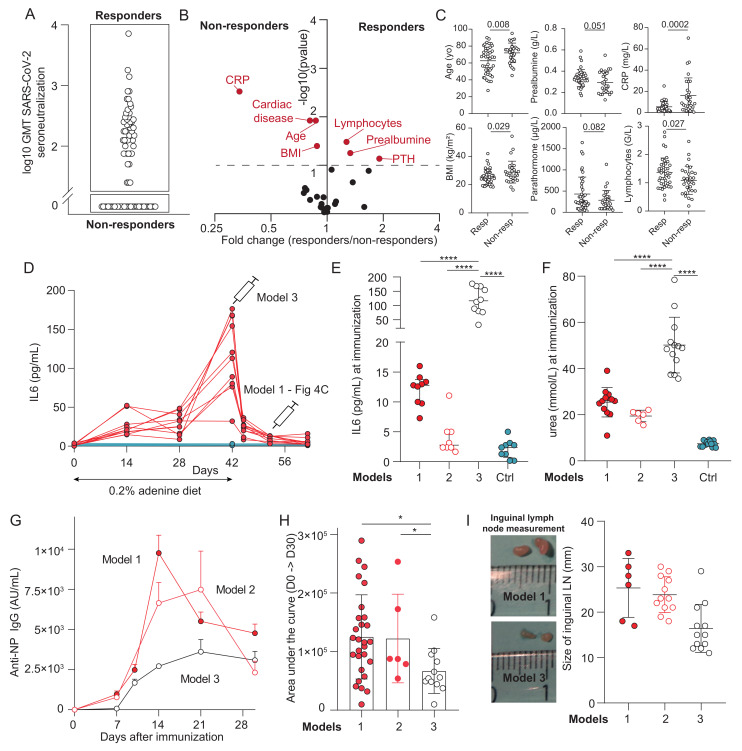
Role of inflammation in the defective humoral response associated with chronic kidney disease. (**A**): Distribution of SARS-CoV-2 serum neutralization capacity in end-stage kidney disease (ESKD) patients after two doses of mRNA vaccine. Patients were distributed into two groups: responders and non-responders. (**B**): Volcano plot displaying the fold changes and statistical significance of the 29 tested clinical and biological variables between responders and non-responders. (**C**): Comparison of the six numerical variables showing differences between responders and non-responders (*p* < 0.1) in univariate analysis and included in the multivariate model. From left to right and top to bottom: age, prealbumin levels, CRP levels, BMI, PTH levels, and lymphocyte count. (**D**): Kinetics of IL-6 concentrations (pg/mL) in mice from the adenine-induced CKD model. The syringe symbols indicate the immunization time points for model 1 (10 days after the end of the adenine regimen) and model 3 (immediately at the end of the regimen). (**E**,**F**): IL-6 levels (**E**) and urea levels (**F**) at immunization compared across the three experimental murine models. Individual values for CKD and control mice are shown, with horizontal lines indicating medians. (**G**): Kinetics of the anti-NP IgG response from day 0 to day 30 following NP-CT immunization in the three CKD models. (**H**): Individual area under the curve (AUC) values for anti-NP IgG responses in mice from the three CKD models. Horizontal lines denote medians. Kruskall–Wallis test. (**I**): Size of the inguinal lymph nodes (LNs) in mice from the three CKD models. Left: representative images. Right: individual LN size measurements, with medians indicated. Kruskall–Wallis test. Abbreviations: CRP, C-reactive protein; IL-6, interleukin-6; AU/mL, arbitrary units per milliliter; IgG, immunoglobulin G; LN, lymph node; GMT, geometric mean titer. Statistical significance: *, *p* < 0.05; ****, *p* < 0.0001.

Despite the lack of association with uremic toxins, ESKD patients displayed markedly reduced anti-RBD IgG responses compared with healthy individuals (Figure 1). To identify the features associated with the humoral defect in ESKD patients, we stratified the latter into “responders” (n = 43/73, 59%) and “non-responders” (n = 30/73, 41%) according to their serum neutralization capacity after two vaccine doses (Figure 5A). Twenty-nine clinical and biological variables were compared between the two groups, encompassing UPLC-measured uremic toxins levels, kidney function markers, nutritional parameters, and immunological indicators (Supplementary Table S2). In exploratory univariate analyses, seven variables—age, BMI, presence of cardiac disease, and levels of CRP, prealbumin, lymphocyte count, and PTH—differed between responders and non-responders (*p* < 0.1; Supplementary Table S2, Figure 5B,C). These variables were subsequently entered into a multivariate model (Supplementary Table S2), which identified CRP level as the only parameter independently associated with the humoral response to vaccination in ESKD patients (OR = 0.86 (0.76–0.96), *p* = 0.015).

To investigate the role of systemic inflammation in the defective humoral response observed in ESKD, we exploited the transient inflammation that occurs at the end of the adenine phase and resolves during the subsequent resting period, while the animals remain in a CKD state. Although the intensity and dynamics of inflammation in this murine model differ from the chronic low-grade profile seen in human CKD—potentially limiting the direct extrapolation of our conclusions—the model uniquely allows us to dissect the effects of inflammation independently of uremic toxins, providing causal insights that are not readily attainable in patients. We, therefore, established a third model (“model 3”) that combines kidney impairment with active inflammation by immunizing mice at the end of the adenine diet (Figure 5D). As expected, IL-6 levels at the time of immunization were markedly elevated in model 3 (60.7 (42.5–98.9) pg/mL) compared with model 1 (11.0 (6.2–15.6); *p* < 0.0001), model 2 (4.8 (3.5–7.1); *p* < 0.0001), and non-CKD controls (4.4 (2.2–10.3); *p* < 0.0001; Figure 5E). Of note, although urea levels were also significantly higher in model 3, the relative difference was much smaller (48.9 (40.5–54.7) mmol/L vs. 26.0 (21.8–28.0), 20.3 (17.0–21.3) for models 1 and 2; *p* < 0.0001; Figure 5F). Strikingly, the humoral response following NP–CT immunization was profoundly impaired in model 3 compared with the two other CKD models, with both peak IgG titers and area under the curve (AUC) values significantly reduced (Figure 5G,H). Importantly, draining inguinal lymph nodes were nearly halved in size in animals exposed to systemic inflammation (model 3) compared with the other groups (Figure 5I). Consistent with these findings, circulating B cell and CD4^+^ T cell counts were also reduced in model 3 compared with non-CKD controls (Supplementary Figure S2A,B,C).

Collectively, our findings indicate that, rather than a direct effect of uremic toxins, systemic inflammation is the primary determinant of the defective humoral response associated with ESKD.

## 3. Discussion

Our translational study, combining an in-depth analysis of an ESKD patient cohort with complementary in vitro and in vivo models, confirms that CKD is associated with an impaired humoral response to protein antigens. Contrary to our initial hypothesis that IS directly affects CD4^+^ T cells and/or B cells, our findings instead indicate that defective antibody generation is primarily driven by CKD-associated inflammation.

Persistent systemic inflammation, reflected by elevated CRP levels, has been consistently reported across numerous clinical and experimental CKD studies, particularly in ESKD [21,22,23]. Chronic inflammation in CKD results from multiple mechanisms within a complex bidirectional relationship [24,25]. Declining eGFR reduces kidney clearance of uremic toxins, including middle-molecule pro-inflammatory cytokines IL-1β, TNF-α, and IL-6 [10]. In parallel, CKD is associated with elevated production of pro-inflammatory cytokines, notably through persistent monocyte activation by toxins such as IS, which signals via the AhR. Interestingly, although numerous studies show that IS activates AhR signaling in monocytes [26,27,28,29], our data suggest that this does not occur in lymphocytes. This difference may reflect the fact that IS is a negatively charged, hydrophilic molecule that cannot passively cross cell membranes and, instead, requires specific transporters for uptake. IS primarily enters cells via organic anion transporters (OATs); however, OAT expression in immune cells remains poorly characterized. Thus, the differential impact of IS on monocytes versus lymphocytes may be due to variations in OAT expression. Regardless, CKD-induced inflammation exacerbates kidney injury, perpetuating a vicious cycle. If CKD-associated inflammation was solely determined by uremic toxin accumulation paralleling eGFR decline, we would expect a perfect positive correlation between uremic toxin levels and defective vaccine responses in our ESKD cohort and CKD mouse model, yet this was not observed. This suggests that, beyond uremic toxins, additional factors contribute to CKD-associated inflammation. These include genetic variability in cytokine promoter regions [30], CKD-associated intestinal dysbiosis [31,32] and periodontal disease [33]. Finally, as CKD progresses, chronic hemodialysis itself may trigger inflammation through translocation of endotoxin induced by intradialytic hypotension [34] and catheter infection [35], as well as through hemodialysis membrane bio-incompatibility [36].

Among the limitations of our study is the relatively small cohort size, which negatively impacts the statistical power and could explain the absence of a detectable correlation between uremic toxin levels and parameters of the humoral immune response in ESKD patients. However, it should be emphasized that this lack of association was consistently observed across both in vitro and in vivo models. Moreover, despite its size, the cohort was sufficient to clearly demonstrate a robust relationship between systemic inflammation and impaired humoral immunity.

Another limitation is the fact that, while our work establishes a strong association between systemic inflammation and impaired humoral immunity, the specific molecular mechanisms by which inflammation disrupts lymphoid architecture and/or impairs lymphocyte function in CKD patients remain undefined. Recent studies have highlighted how lymphoid stromal networks and chemokine gradients, such as CXCL13 produced by reticular cells, are essential for B cell zone organization and effective humoral responses [37]. Sustained inflammatory cytokine signaling (e.g., IL-6) has been implicated in altering stromal cell homeostasis, which could disrupt fibroblastic reticular cell niches and chemokine gradients required for germinal center maintenance [38]. In line with this idea, the marked lymph node shrinkage in the inflammatory CKD mouse model, which was not observed in non-inflamed CKD mice, is reminiscent of the structural disruptions reported in other inflammatory states, including sepsis and sterile inflammation [39]. Such architectural disorganization has been shown to impair antigen retention and presentation, ultimately weakening B cell responses in experimental models [39].

Chronic inflammation-associated impairment of adaptive immunity is not unique to CKD, and evidence from other disease contexts may inform the CKD setting. Inflammation-induced immunosuppression has been particularly well-studied in sepsis [40], where it is recognized as a multifactorial process, characterized by the expansion of regulatory immune populations [41,42,43] and by the depletion of effector lymphocytes. Consistent with this theory, we confirmed marked lymphopenia in our CKD cohort, the severity of which was associated with impaired vaccine responses. A recent large cohort study, including CKD patients, further established a strong positive correlation between the CRP/lymphocyte ratio and CKD [44]. Furthermore, although the retrospective nature of our study did not allow for assessing regulatory populations in ESKD patients, other reports have demonstrated elevated levels of myeloid-derived suppressor cells in this population [45,46].

Another thoroughly explored pathological setting that mirrors the coexistence of immune insufficiency and chronic inflammation is inflammaging [47]. Inflammaging promotes immune deficiency through persistent, low-grade inflammatory signaling that progressively erodes immune cell function and renewal. Chronic exposure to proinflammatory cytokines (IL-6, TNF, IL-1β), driven by senescent cells and their senescence-associated secretory phenotype (SASP), induces T cell exhaustion, impairs antigen presentation, and skews hematopoiesis toward dysfunctional myeloid lineages [48,49]. Sustained activation of NF-κB and cGAS–STING pathways by DNA damage and cytosolic nucleic acids leads to maladaptive innate immune activation and interferon desensitization, blunting antimicrobial responses [50]. Concurrent metabolic dysregulation, including mitochondrial dysfunction, NAD^+^ depletion, and mTOR hyperactivation, compromises immune cell fitness and longevity [51]. Age-related impairment of macrophage efferocytosis and gut microbiota dysbiosis further perpetuate chronic inflammation while weakening immune resolution and immune education [52]. Together, these interconnected mechanisms create an immune system that is chronically inflamed, yet functionally incompetent, resulting in increased susceptibility to infection and poor vaccine responses, mirroring the situation observed in CKD [53,54].

In conclusion, our data suggest that the humoral defect in CKD patients is primarily a consequence of chronic inflammation, driven, in part, by uremic toxins acting on innate immunity (rather than directly on adaptive immune cells, as we initially suspected), but also by additional patient- and treatment-related factors. This inflammatory milieu, in turn, induces adaptive immune paralysis through depletion of effector lymphocytes and disruption of lymphoid architecture, mirroring what is observed in other chronic inflammatory conditions. Whether targeting inflammation in CKD patients—as tested to reduce inflammation-induced cardiovascular mortality in dialysis patients [55]—would also help in restoring immune competence, improving vaccine responsiveness and ultimately reducing infectious complications, is an attractive hypothesis that remains to be confirmed in future interventional studies.

## 4. Materials and Methods

### 4.1. Cohort of ESKD Patients and HVs

The patient cohort consisted of individuals with end-stage kidney disease (ESKD), undergoing hemodialysis in two dialysis centers at the Lyon University Hospital, who had received two doses of mRNA anti-SARS-CoV-2 vaccine. The healthy volunteer (HV) cohort comprised healthcare professionals vaccinated during the same period with the same vaccination regimen. All participants were sampled between 10 and 14 days after the second vaccine injection for analysis of humoral and cellular responses. This study was conducted in accordance with French legislation on biomedical research and the Declaration of Helsinki, and the protocol was approved by a national ethical research committee (ID-RCB 2021-A00325-36).

### 4.2. Assessment of Anti-SARS-CoV-2 Immune Responses

The serological response was assessed by quantification of anti-RBD IgG based in a chemiluminescence technique, using the Maglumi SARS-CoV-2 S-RBD IgG test (Snibe Diagnostic, Shenzhen, China) on a Maglumi 2000 analyzer (Snibe Diagnostic, Shenzhen, China), according to the manufacturer’s instructions, and obtained titers were expressed as binding arbitrary units/mL (BAU/mL).

Live virus neutralization assay consisted in the incubation of 2.5 × 10^4^ VeroE6cells with 200PFU of SARS-CoV2 (BetaCoV/France/IDF0571/2020 virus [Global Initiative on Sharing Avian Influenza Data Accession ID = EPI_ISL_411218], kindly provided by Dr O. Terrier) in the presence of successive dilution of sera (from 1:100 to 1:12,800) made in quadruplicate, as previously described [9]. After 5 days’ incubation, the viral cytotoxicity was revealed by crystal violet staining, and results were expressed as the inverse of the geometric mean titers of the lowest dilution which protected against viral cytotoxicity.

The spike specific cellular CD4+ response was assessed by a commercial Interferon-gamma-releasing assay (IGRA), the QuantiFERON^®^ SARS-CoV2 test (Qiagen, Venlo, The Netherlands), in which 1 mL of fresh blood was incubated with 13 mers peptides derived from SARS-CoV-2 spike glycoprotein, and with a negative control (uncoated tube) for background noise. After 20 h of culture at 37 °C, interferon gamma was quantified in the supernatants by ELISA and expressed in IU/mL as the difference between the value of the CD4+ tube and the uncoated tube.

The percentages of spike specific follicular helper T cells (LTfh) were quantified as in references [9,56]. Briefly, 10^6^ PBMCs were incubated with 1 µg/mL of a peptide pool overlapping the spike glycoprotein (PepMixTM, JPT Peptides Technologies GmbH, Berlin, Germany). After 16 h, cells were rinsed and marked by a panel of fluorescent antibodies composed of CD4 (SK3) from Biolegend, San Diego, CA, USA, as well as CD3 (UHCT1), CXCR5 (RF8B2), and CD25 (2A3), from BD Biosciences, Franklin Lakes, NJ, USA. Cells were fixed with 2% methanol-free formaldehyde. Sample acquisitions were made on a BD LSR Fortessa 4L flow cytometer (BD Biosciences, Franklin Lakes, NJ, USA).

### 4.3. Allogeneic Cocultures

For the B cell proliferation assays, CD4+ T cells and B cells were purified (up to 95% purity) from PBMCs collected in healthy donors by negative selection with magnetic enrichment kits (R&D Systems, Minneapolis, MN 55413, USA). B cells were stained with a proliferation dye (CellTrace Violet, Thermo Fisher Scientific, Waltham, MA, USA) according to the manufacturer’s instructions. Thereafter, 4 × 10^5^ B cells were cocultured with 4 × 10^4^ allogeneic CD4+ T cells in the presence of a soluble anti-human IgM F(ab′)2 (5 µg/mL; Jackson ImmunoResearch, Cambridge, UK). Cells were cultured in a complete culture medium composed of RPMI 1640 GlutaMAX medium (Invitrogen, Carlsbad, CA, USA), supplemented with 10% fetal calf serum, 25 mM Hepes (Invitrogen, Carlsbad, CA, USA), and penicillin/streptomycin (10 U/mL; Invitrogen, Carlsbad, CA, USA). After 6 days of culture at 37 °C, cells were rinsed and incubated at 4 °C with the following fluorescent antibodies: CD3 (UHCT1), CD4 (SK3), CD19 (HIB19), and CD20 (2H7), all from BD Biosciences (Franklin Lakes, NJ, USA), and a Fixable Viability Dye from eBioscience (Thermo Fisher Scientific, Waltham, MA, USA. Samples were analyzed on a BD LSR Fortessa 4L flow cytometer (BD Biosciences, Franklin Lakes, NJ, USA).

Indoxyl sulfate (Thermo Fisher Scientific, Waltham, MA, USA) at 40µM was added to the complete culture medium at the indicated time. Experiments used for quantification of free IS in the complete medium (RPMI medium supplemented with 10% fetal calf serum) were based on fluorometric determination of IS value based on an excitation wavelength at 280 nm and an emission wavelength of 383 nm.

### 4.4. Quantification of Uremic Toxins by Ultra-High-Performance Liquid Chromatography (UPLC) and Ultraviolet and Fluorescence Detection

Plasma samples were heat deproteinized, centrifuged and filtered using Amicon Ultra 0.5 mL Filters (molecular weight cut-off 30 kDa, Merck KGaA, Darmstadt, Germany) to determine the total toxin concentration, while determination of the free fraction started with an Amicon filtration step. The obtained ultrafiltrate was analyzed on an Agilent 1290 Infinity device (Agilent, Santa Clara, CA, USA). Chromatographic separation was performed at 26 °C on a Waters Acquity UPLC BEH C18 column (1.7 µm, 100 × 2.1 mm) with a Waters Acquity UPLC BEH C18 Van Guard column (1.7 µm, 5 × 2.1 mm). The mobile phase consisted of a 50 mM ammonium formate buffer (mobile phase A, pH 3.0) and methanol (mobile phase B). A linear gradient elution was used to separate the compounds at a flow rate of 0.3 mL/min, and started at 98% A, followed by a composition change to 90% A in 7 min. In the next 9 min, the mobile phase changed to 100% B and was held for 3 min. Uric acid, hippuric acid, and CMPF were detected with an Agilent G4212A diode array detector at 300 nm, 245 nm, and 254 nm respectively. Indoxyl sulfate (λex: 280 nm, λem: 376 nm), p-cresyl sulfate and p-cresyl glucuronide (λex: 264 nm, λem: 290 nm), and indole-3-acetic acid (λex: 280 nm, λem: 350 nm) were detected by an Agilent G1316C fluorescence detector, as previously described [57].

### 4.5. Mice Experiments

Eight- to ten-week-old C57/Bl6J female mice (from Charles River Laboratories, Wilmington, DE, USA) were used in all experiments. Mice were housed in a pathogen-free barrier facility with 12 h light/dark cycle and ad libitum access to water and food in standard animal care facility rooms at 21 °C. All studies and procedures were performed in accordance with European Union guidelines and were approved by the local ethical committee for animal research (CECCAPP SFR Biosciences, Gerland, France, UMS3444/US8, Lyon, registered by the French National Ethics Committee of Animal Experimentation). The ethics approval number for this protocol is APAFIS#24971-2020022410571420.

#### 4.5.1. Models 1 and 3: Adenine-Induced CKD

Mice were fed with 0.2% (*w*/*w*) adenine-enriched food (SAFE, Augy, France) for 6 weeks. This regimen was interspersed with two 3-day periods (from d12 to d15 and from d26 to d29) in order to avoid an excessive weight loss due to decreased food consumption during adenine diet. At the end of the regimen, mice were fed with standard diet for the rest of the experiment. The control group were fed with standard diet during the entire experiment.

#### 4.5.2. Model 2: 5/6 Nephrectomy

The surgery consisted of two consecutive steps: (1) initial resection of the upper and lower pole of the right kidney during the first surgery, and (2) left kidney resection one week after the first surgery. All operating procedures were performed under aseptic techniques. Mice were anesthetized with ketamine/xylazine mixture injected intraperitoneally (100/20 µg/g) and placed on a warm pad during the surgery. In the supine position, a midline abdominal incision was made, and the right kidney was exposed. The surgeon performed careful separation of perikidney fat, connective tissue, the adrenal gland, and the ureter with blunt forceps. Upper and lower poles were resected with a soldering iron to limit bleeding. After checking that there was no bleeding, the abdominal muscle layer and skin were closed with 5–0 sterile non-absorbable sutures, using a simple continuous technique. Subsequently, the contralateral left kidney Nx was performed 7 days after the first operation. The Nx was performed by reopening the previous midline laparotomy incision, and the left kidney was isolated. Care was taken to preserve the adrenal gland, after which the kidney pedicle, with the artery, vein, and ureter, was ligated. Finally, the kidney was extirpated by transecting the vessels and ureter immediately distal to the ligature. The abdominal muscle layer and skin were closed with 5–0 sterile non-absorbable sutures, using a simple continuous technique. For antalgic, buprenorphine (50 µg/g body weight) was injected SC 15 min before the surgery, and every 12 h after the surgery for 48 h, for a total of 4 injections. The sham surgery group also underwent two-step surgeries, but without pole resection of the right kidney or left kidney resection.

#### 4.5.3. Immunization and Sampling

The used immunogen was the hapten NP coupled with the choleric toxin; 1 µg of NP-CT reconstituted in 50 µL of sterile 1X phosphate-buffered saline was injected subcutaneously at the tail base of mice at the indicated time points. Mice were blood-sampled every week during the experiments.

#### 4.5.4. Quantification of Urea, IL6 and Humoral Response

Urea was quantified in sera of mice by a Urea Nitrogen colorimetric detection kit (ThermoFisher Scientific), following the manufacturer instructions.

Interleukine-6 was quantified by the IL-6 Mouse ProcartaPlex™ Simplex Kit, High Sensitivity (InVitrogen, ThermoFisher Scientific): briefly, sera were incubated with fluorescent capture microbeads precoated with anti-IL6 specific biotinylated antibody, subsequently marked by fluorescent streptavidin. Bead fluorescence was quantified on a MAGPIX Luminex reader (ThermoFisher Scientific) at the indicated wavelength.

For specific anti NP response, we quantified anti-NP IgG by standard ELISA: briefly, Maxisorp plates were coated overnight at 4 °C with NP(25)-BSA (0.15 µg/mL, 100 µL/well). After blocking with 2% BSA in PBS, serum samples were serially diluted and incubated for 1.5 h at 37 °C. Alkaline phosphatase-conjugated anti-IgG (1:2000) was added, followed by substrate (phosphate substrate in diethanolamine buffer, pH 9.8). Absorbance was read after 8 min. All incubations were at 37 °C, with PBS-T washes between steps.

### 4.6. Statistical Analysis

The normality of data distribution was assessed using the Shapiro–Wilk test. Normally distributed variables are expressed as mean ± standard deviation (SD) and were compared using the unpaired or paired Student’s *t*-test for two groups, or one-way ANOVA with Tukey’s post hoc test for multiple comparisons. Non-normally distributed data are presented as median with interquartile range (IQR) and were analyzed using the Mann–Whitney U test for independent samples or the Wilcoxon signed-rank test for paired samples. For multiple comparisons of non-normal data, the Kruskal–Wallis test, followed by Dunn’s post hoc test, was applied. For multiple comparisons of linear regression data made in Figure 3E, we applied a Bonferroni correction. Categorical variables were compared using the chi-square or Fisher’s exact tests, as appropriate. Statistical analyses for identification of variables associated with vaccine response in ESKD patients (Figure 5B and Table S2) were conducted using univariate and multivariate logistic regressions with R software (version 4.3.0), including variables with a *p*-value < 0.10 in the univariate analysis for the multivariate model. Other statistical analyses were performed using GraphPad Prism v8.0, and statistical significance was defined as *p* < 0.05.

## Data Availability

The original contributions presented in this study are included in the article/Supplementary Materials. Further inquiries can be directed to the corresponding author.

## Supplementary Materials

The following supporting information can be downloaded at: https://www.mdpi.com/article/10.3390/toxins18020104/s1, Figure S1: Experimental setup and quantification of free indoxyl sulfate for coculture experiments; Figure S2: Alterations in Blood B-Cell and CD4^+^; T-Cell Populations in CKD Mouse Models. Table S1: Clinical characteristics of the cohorts; Table S2: Univariate and multivariate analysis of variables associated with vaccine response in End-Stade Kidney Disease patients.
