# Supplementary material for: Chronic Kidney Disease-Associated Defect in Humoral Immune Response Is Driven by Inflammation
Source: Toxins (Basel). 2026 Feb 19;18(2):104. doi: 10.3390/toxins18020104 (PMC12944840; doi:10.3390/toxins18020104)
Supplement: Supplementary file 1 [file toxins-18-00104-s001.zip › toxins-4080910-supplementary.pdf]

Supplementary Table S1 – Clinical characteristics of the cohorts

		HV (n=26)	ESKD (n=80)
**Gender (F/M)**		13/13	30/40
**Age (years old)**		53 [31-59]	68 [56-77]
**BMI (kg/m²)**		23 [21-25]	26 [23-30]
**Comorbidities**			
	Cardiac	1 (4)	40 (50)
	Respiratory insuffisiency	0 (0)	10 (12.5)
	Diabetes	0 (0)	35 (44)
	Smoking	3 (12)	11 (14)
**HD parameters**			
	Time in dialylsis (days)	na	1158 [482-2023]
	AVF	na	56 (70)
	Diurese > 250cc/d	26	33 (41)

Abbreviations are: HV: healthy volunteers; ESKD: End Stage Kidney Disease patients treated with maintenance hemodialysis; BMI: body mass index; HD: hemodialysis AVF: arterio venous fistula

Supplementary Table S2 – Univariate and multivariate analysis of variables associated with vaccine response in End-Stade Kidney Disease patients.

Variable	Responders (n=43)	Non-responders (n=30)	Univariate			Multivariate		
			OR	95% CI	P value	OR	95% CI	P value
**Demographics**								
Age (years)	62.6 +/-16.1	71.9 +/- 11.6	0,95	[0,92-0,99]	0.012	0,97	[0,92-1,02]	0,257
BMI (kg/m²)	25.5 +/- 5.3	28.9 +/- 7.7	0,92	[0,84-0,99]	0.040	0,98	[0,87-1,11]	0,782
**Comorbidities**								
Time in dialysis (days)	2016 +/-2484	1206 +/-1009	1	[1-1]	0.130			
Diabetes	19 (44.2%)	12 (40%)	1,19	[0,46-3,1]	0.722			
Cardiac disease	17 (39.5%)	21 (70%)	0,28	[0,1-0,74]	0.012	0,43	[0,11-1,55]	0,197
Pulmonary disease	3 (7%)	4 (13.3%)	0,49	[0,09-2,38]	0.372			
Hepatic disease	0 (0%)	3 (10%)	NA	[NA-NA]	0.990			
**Biological datas**								
Hemoglobine (g/L)	110.0 +/- 13.3	109 +/-13.6	1,01	[0,97-1,04]	0.756			
CRP (mg/L)	5.5 +/-4.7	16.2 +/- 16.5	0,88	[0,79-0,94]	0.003	**0,87**	**[0,76-0,96]**	**0,015**
Albumine (g/L)	36.8 +/-5.2	34.8 +/-5.3	1,08	[0,98-1,2]	0.123			
Prealbumine (g/L)	0.4 +/-0.4	0.3 +/-0.1	267	[1,64-123293]	0.056	7,41	[0,96-1778]	0,176
Lymphocytes (G/L)	1.4 +/-0.5	1.1 +/-0.5	3,12	[1,17-9,74]	0.033	2,15	[0,62-9,14]	0,256
Monocytes (G/L)	0.6 +/-0.2	0.6 +/-0.3	1,38	[0,18-11,5]	0.760			
Phosphore (mM)	1.5 +/-0.5	1.5 +/- 0.4	1,05	[0,4-2,84]	0.917			
Predialytic urea (mM)	20.0 +/- 5.4	20.3 +/-6.2	0,99	[0,91-1,08]	0.822			
Posdialytic urea (mM)	4.9+/- 4.4	5.3 +/- 3.5	0,98	[0,86-1,11]	0.727			
PTH (ng/L)	526.4 +/-741.8	275.8 +/- 231.3	1	[1-1]	0.073	1	[1-1]	0,669
**Uremic toxins**								
Total IS (µM)	102.6 +/-50.5	104.7 +/-52.1	1	[0,99-1,01]	0.865			
Free IS (µM)	7.651 +/- 7.681	8.171 +/-5.460	0,99	[0,92-1,06]	0.747			
Total pCS (mM)	1.153 +/- 0.717	1.233 +/-0.690	0,85	[0,43-1,67]	0.631			
Free pCS (mM)	0.120 +/- 0.095	0.136 +/-0.063	0,11	[0-32,4]	0.445			
Total pCG (mM)	0.218 +/- 0.264	0.281 +/-0.271	0,41	[0,06-2,39]	0.324			
Free pCG (mM)	0.192 +/- 0.238	0.252 +/-0.245	0,35	[0,04-2,46]	0.295			
Total IAA (mM)	0.180 +/- 0.228	0.222 +/- 0.323	0,56	[0,08-0]	0.517			
Free IAA (mM)	0.055 +/- 0.080	0.057 +/- 0.082	0,76	[0-590]	0.925			
CMPF (mM)	0.357 +/-0.356	0.260 +/-0.275	2,85	[0,61-19,3]	0.224			
Total HA (mM)	4.378 +/-3.401	3.919 +/-3.237	1,04	[0,91-1,21]	0.559			
Free HA (mM)	2.484 +/-2.130	2.250 +/-1.903	1,06	[0,84-1,35]	0.627			
Uric acid (mM)	6.689 +/-1.604	6.993 +/-1.884	0,9	[0,67-1,18]	0.455			

Abbreviations are: BMI : body mass index; CRP: C reactive protein; PTH : parathormone ; IS: Indoxyl sulfate; pCS, p-cresylsulfate; pCG, p-cresylglucuronide; IAA, indole acetic acide; CMPF, 3-Carboxy-4-methyl-5-propyl-2-furanpropionic acid; HA, hippuric acide, mM, mmol/L; µM, µmol/L, OR, odds ratio; 95 CI, 95% confident interval

Supplementary Figures Legends:

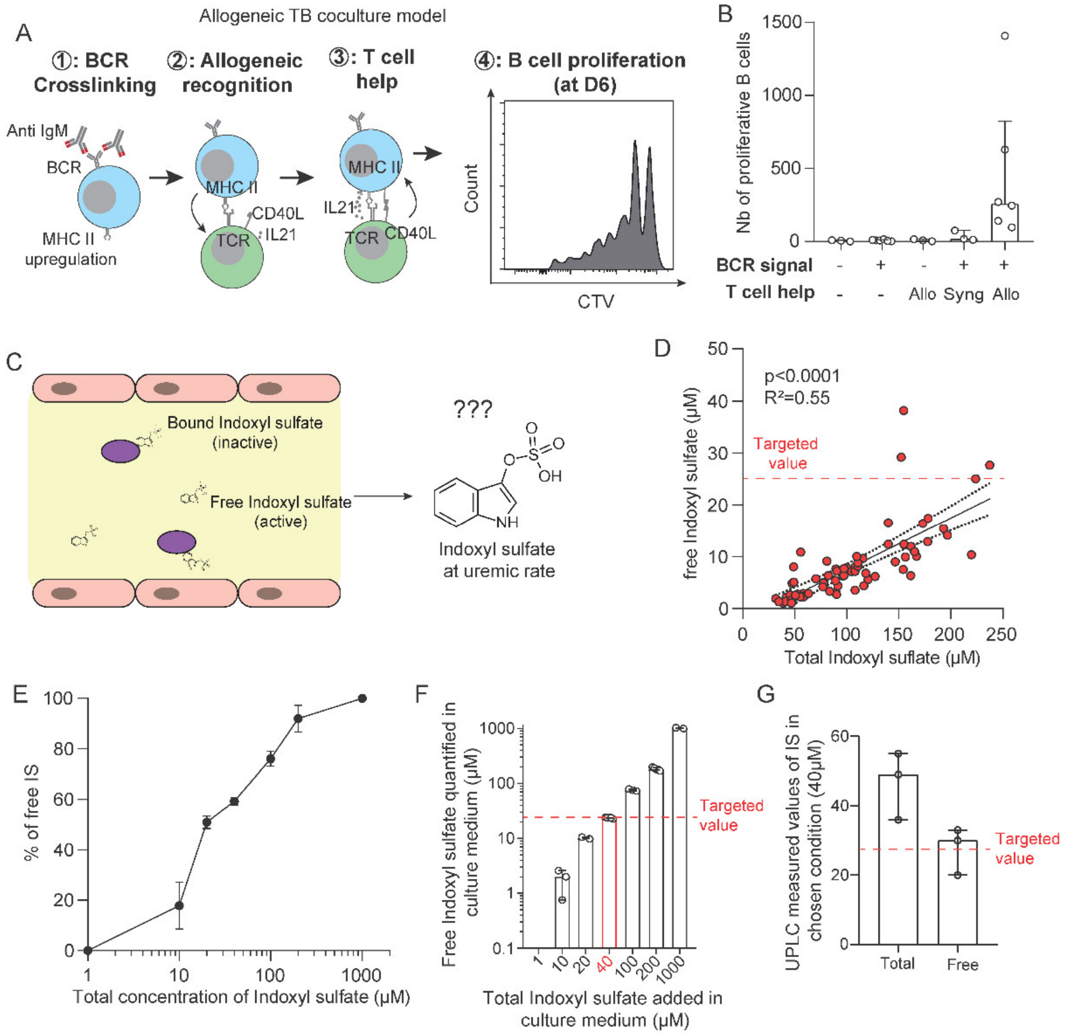

Supplementary Figure S1: Experimental setup and quantification of free indoxyl sulfate for coculture experiments.

(A) Schematic representation of the allogeneic T–B coculture system. B cells are stimulated via B cell receptor (BCR) crosslinking (anti-IgM), which induced upregulation of MHCII on B cell. The TCR of B cell recognize the allogeneic MHCII of B cell, leading to T cell activation and upregulation of costimulatory signals (CD40L and IL21). The activated T cell provided costimulatory signal to the B cell. Proliferation of B cells is assessed at day 6 (D6) using CellTrace Violet (CTV). Representative histogram of B cell proliferation (CTV dilution) is shown.

(B) Number of proliferative B cells in coculture conditions, with or without BCR crosslinking and/or T cell help. Allo : Allogeneic Tcell, Syng: syngenic T cell

(C) Illustration of the equilibrium between protein-bound (inactive) and free (active) indoxyl sulfate in serum in the uremic context.

(D) Relation between total and free indoxyl sulfate concentrations in serum samples from ESKD patients (n = 74). The red dashed line indicates the targeted free indoxyl sulfate value for in vitro experiments.

(E) Percentage of free indoxyl sulfate as a function of total indoxyl sulfate dose added to the coculture medium.

(F) Measured free indoxyl sulfate concentrations in culture medium supplemented with increasing doses of total indoxyl sulfate. The red dashed line indicates the targeted free indoxyl sulfate value.

(G) UPLC-measured total and free indoxyl sulfate concentrations in the chosen experimental condition (40 µM total IS). The red dashed line indicates the targeted free indoxyl sulfate value.

Supplementary Figure S2: Alterations in Blood B-Cell and $CD4^{+}$ T-Cell Populations in CKD Mouse Models.

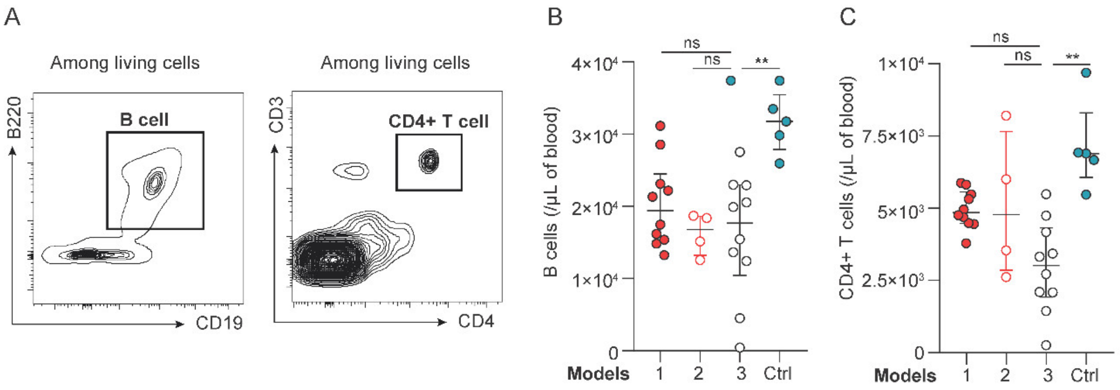

(A) Representative flow cytometry plot showing the gating strategy for B cells (CD19+B220+) and CD4+ T cells (CD3+CD4+) quantification.

The number of B (B) and CD4+ T (C) lymphocytes was quantified in the blood of control (blue) animals and mice from the 3 CKD models (Model 1: adenine diet, red dots; Model 2: 5/6 nephrectomy, red open circle; Model 3: adenine diet with inflammation, black open circle) by flow cytometry. Individual data are plotted with mean ± SEM. Kruskall-Wallis test. **, $p < 0.01$; ns = non-significant.
